# Draft Genome Sequence of a Caffeine-Utilizing Bacterium, *Cupriavidus* sp. Strain D384

**DOI:** 10.1128/genomeA.00370-17

**Published:** 2017-05-18

**Authors:** Saori Watahiki, Nobutada Kimura

**Affiliations:** a Bioproduction Research Institute, National Institute of Advanced Industrial Science and Technology (AIST), Tsukuba, Japan; b Graduate School of Life and Environmental Sciences, University of Tsukuba, Tsukuba, Ibaraki, Japan

## Abstract

*Cupriavidus* sp. D384 was isolated from forest soil in Japan and is known to utilize caffeine as a sole source of carbon and energy. We report here the 6,835,230-bp genome sequence for this strain, which contains 6,116 predicted coding sequences, including gene operon for alkaloid degradation.

## GENOME ANNOUNCEMENT

Caffeine (1,3,7-trimethyl xanthine) is an alkaloid and member of the xanthine family of compounds. It is known that caffeine is present in a wide range of environments, such as wastewater, ground water, and remote mountain lakes (1, 2). *Cupriavidus* sp. strain D384 was isolated from forest soil in Tsukuba, Japan, and shown to act as a caffeine degrader by utilizing caffeine as a sole source of carbon and energy. Whole-genome shotgun sequencing on strain D384 was performed by the Hokkaido System Science Co., Ltd. using a shotgun sequencing method with paired-end sequencing on a HiSeq 2000 sequencing system (Illumina), followed by assembly with Velvet version 2.6. The draft genome is 6,841,708 bp in size and contains 84 contigs with an average contig length of 81.4 kb, an *N*_50_ of 298,055, and an average G+C content of 64.9 mol%.

The genome sequence of strain D384 was uploaded to the RAST server (http://rast.nmpdr.org) to predict the open reading frames (ORFs), tRNAs, and rRNAs (3). Rapid genome annotation using the RAST annotation server revealed 6,116 coding sequences (CDSs), 56 tRNA sequences, and 3 rRNA sequences. The coding sequences were classified into 4,506 subsystems, the most abundant of which were for metabolism of amino acid derivatives (*n* = 634 CDSs) and carbohydrates (*n* = 563); metabolism of cofactors, vitamins, prosthetic groups, and pigments (*n* = 368); membrane transport (*n* = 375); protein metabolism (*n* = 287); stress response (*n* = 199); and construction of the cell wall and capsule (*n* = 164). A comparison of *Cupriavidus* sp. strain D384 with other *Cupriavidus* strains within the RAST database identified *C. metallidurans* strain CH34 (Genome ID266264.4) as its closest neighbor, with a score of 515, followed by *C. taiwanensis* (Genome ID164546.7), with a score of 488. *C. pinatubonensis* JMP134 (Genome ID264198.3) was the third closest neighbor, with a score of 488. *C. metallidurans* CH34 is known as a metal-resistant bacterium (4).

The comparison of the functional annotation was performed using the Kyoto Encyclopedia of Genes and Genomes (KEGG) database (5). Strain D384 possessed the ring-cleavage dioxygenase, 2-hydroxy-6-oxo-6-phenylhexa-2,4-dienoate hydrolase, and 2-keto-4-pentenoate hydratase of biphenyl degradation; whole benzoate degradation via the hydroxylation pathway; and phenol hydroxylase components of the phenol degradation pathway.

### Accession number(s).

The whole-genome sequence of *Cupriavidus* sp. strain D384 has been deposited in DDBJ/EMBL/GenBank under the accession numbers BBQI01000001 to BBQI01000134.
